# Vibriocidal activity of certain medicinal plants used in Indian folklore medicine by tribals of Mahakoshal region of central India

**DOI:** 10.4103/0253-7613.55212

**Published:** 2009-06

**Authors:** Anjana Sharma, Virendra Kumar Patel, Animesh Navin Chaturvedi

**Affiliations:** Bacteriology Laboratory, Department of Post Graduate Studies and Research in Biological Sciences, R.D. University, Jabalpur (M.P.) 482001, India

**Keywords:** Disk diffusion method, Medicinal plants, minimum inhibitory concentration, Vibriocidal activity

## Abstract

**Objectives:**

Screening of the medicinal plants and determination of minimum inhibitory concentration (MIC) against *Vibrio cholerae* and *Vibrio parahaemolyticus*.

**Materials and Methods:**

A simple *in vitro* screening assay was employed for the standard strain of *Vibrio cholerae*, 12 isolates of *Vibrio cholerae* non-O1, and *Vibrio parahaemolyticus*. Aqueous and organic solvent extracts of different parts of the plants were investigated by using the disk diffusion method. Extracts from 16 medicinal plants were selected on account of the reported traditional uses for the treatment of cholera and gastrointestinal diseases, and they were assayed for vibriocidal activities.

**Results:**

The different extracts differed significantly in their vibriocidal properties with respect to different solvents. The MIC values of the plant extracts against test bacteria were found to be in the range of 2.5-20 mg/ml.

**Conclusions:**

The results indicated that *Lawsonia inermis, Saraca indica, Syzygium cumini, Terminalia belerica, Allium sativum*, and *Datura stramonium* served as broad-spectrum vibriocidal agents.

## Introduction

Cholera is an acute intestinal disease caused by *Vibrio cholerae*. The organism has a short incubation period of less than a day, extending up to five days. The enterotoxin produced by this rod causes copious, painless, watery diarrhea leading to vomiting, severe dehydration, and even death if treatment is not prompt.[1] Cholera can spread as an endemic, epidemic, or pandemic. Typically, antimicrobial agents are administered for 3-5 days; however, a single-dose therapy with tetracycline, doxycycline, furazolidone, or ciprofloxacin has been seen to be effective in reducing the duration and volume of diarrhea. However, the resistance to antimicrobial agents, driven in part by their widespread traditional use, has become an increasing problem throughout the world.[2]

With the alarming incidence of antibiotic resistance in bacteria of medical importance,[3] there is increasing interest in plants as a source of agents for the treatment of microbial diseases. According to the World Health Organization more than 80% of the world's population relies on traditional medicine for their primary healthcare needs.[4] The most significant bioactive compounds obtained from plants include alkaloids, flavonoids, tannins and phenolic substances.[5] The vibriocidal action of different extracts from the leaves of the shrub *Punica granatum* was studied against various strains of *Vibrio cholerae*.[6] The leaf extract of *Mimusaops elengi* showed antibacterial activity against *V. cholerae* and other bacteria.[7] Muruganandan *et al.* have reported the antibacterial activity of the *Syzygium cumini* bark.[8] Reddy *et al*. have found antibacterial constituents from the berries of *Piper nigrum*.[9]

A justified belief persists that traditional medicine is a lot cheaper and more effective than modern medicine. In developing countries, people belonging to the poor socioeconomic background use folk medicine for the treatment of common infections.[10] Research on bioactive substances could possibly lead to the discovery of new compounds, resulting in the formulation of new and more potent antibacterial drugs, to overcome the problem of resistance to currently available antibiotics. This article reports the results of a survey that was done based on folk uses by traditional practitioners in the tribal area of the Mahakoshal region of Central India, along with a bioassay test for vibriocidal activity. In the present study a total of 16 plants were studied to determine and analyze the vibriocidal activity.

## Materials and Methods

### Collection and preparation of plant material

The plant samples were collected from tribal regions of Mandla and Dindori (M.P.), India; based on the information provided in the enthnobotanical survey of India and from the local medicine men of tribal regions, and were screened for potential vibriocidal activity [Table 1].

**Table 1:** Medicinal plants screened for potential vibriocidal activity

*Botanical name*	*Family*	*Vernacular name*	*Parts used*
*Syzygium cumini* (Linn.) Skeels.	Myrtaceae	Jamun	Bark (stem)
*Lawsonia inermis* Linn.	Apocynaceae	Mehndi, Henna	Leaf
*Terminalia belerica* Roxb.	Combretaceae	Baehra	Fruit
*Saraca indica* Linn.	Caesalpinaceae	Ashoka	Bark (stem)
*Datura stramonium* Linn.	Solanaceae	Datura	Leaf
*Allium sativum* Linn.	Liliaceae	Lahsun	Bulbs
*Madhuca latifolia* Roxb.	Sapotaceae	Mahua	Bark (stem)
*Acacia catechu* (Linn.) Willd.	Mimosoidae	Khair	Bark (stem)
*Acacia arabica* (Lamk.) Willd.	Mimosaceae	Babool	Bark (stem)
*Azadirachta indica* Juss.	Meliaceae	Neem	Leaf
*Tamarindus indica* Linn.	Saptaceae	Imli	Leaf
*Punica granatum* Linn.	Punicaceae	Anar	Leaf
*Eugenia caryophyllus* Spreng.	Myrtaceae	Laung/clove	Flower buds
*Mimusops elengi* Linn.	Sapotaceae	Maulsari	Leaf
*Mangifera indica* Linn.	Anacardiaceae	Aam	Leaf
*Piper nigrum* Linn.	Pipraceae	Kalimirch	Fruit

### Source of microorganisms

The microorganisms were obtained from the culture collection center of the Bacteriology Laboratory, R. D. University, Jabalpur, and (NICED), Kolkata. A total of 12 bacterial strains of *Vibrio cholerae* non-O1 (BGCC#59, BGCC#60, BGCC#61, BGCC#62, BGCC#63, BGCC#64, BGCC#65, BGCC#66, BGCC#67, BGCC#68, BGCC#69, and BGCC#70), involved in the present study were obtained from the culture collection center of the Bacteriology Laboratory, R.D. University, Jabalpur (MP), India. Standard strains of *Vibrio cholerae* (H17004) and *Vibrio parahaemolyticus* (KX-V138) were obtained from the National Institute of Cholera and Enteric Diseases (NICED), Kolkata.

### Inoculum preparation of bacterial cultures

The method of Collins *et al*.,[11] was followed with certain modifications for the preparation of bacterial inoculum. Accurately 0.2 ml of overnight cultures of each organism were dispensed into 20 ml of sterile nutrient broth and incubated at 37°C for 24 hours to standardize the culture to 10^6^ cfu/ml. A loopful of this standardized culture was used for the antibacterial assay.

## Preparation of Plant Extracts

### Aqueous extraction

Ten grams of air-dried powder was placed in distilled water and boiled for 6 hours. At intervals of 2 hours it was filtered through eight layers of muslin cloth and centrifuged at 5000 X g for 15 minutes and the supernatants were collected. The supernatant was concentrated for 6 hours to make the final volume one-fourth of the original volume.[12]

### Solvent extraction

Ten grams of air-dried powder was placed in 100 ml of organic solvent (ethanol and acetone) in a conical flask, plugged with cotton, and kept on a rotary shaker at 190-220 rpm for 24 hours. After 2 hours it was filtered through eight layers of muslin cloth and centrifuged at 5000 X g for 15 minutes. The supernatants were collected and the solvents were evaporated to make the final volume one-fourth of the original volume.[12]

### Antibacterial assays

Vibriocidal activity was tested using the disk diffusion method.[13] Petri plates containing 20 ml of Mueller Hinton agar medium were seeded with a 24-hour old culture of bacterial strains. The plant extracts were tested in a concentration of 40 mg/ml, applying 10 μl of each sample to sterile filter paper disks (5 mm in diameter) and placed on the surface of the medium. Incubation was performed at 37°C for 24 hours. The inoculum size was adjusted to achieve a final inoculum of approximately 10^8^ cfu/ml. The assessment of vibriocidal activity was based on the size of the inhibition zone formed around the disk.

### Determination of minimum inhibitory concentration of plant extracts against test bacteria

The minimum inhibitory concentration (MIC) of the plant extracts were determined by the modified methods of Rios *et al.*[13] and Rojas *et al.*[10] The test was performed for a 2.5 mg/ml – 20 mg/ml concentration range (2.5, 5, 10, 15, 20 mg/ml) of each plant extract. The disks were impregnated with 10 μl of plant extracts, of different concentrations, placed on the Mueller Hinton inoculated agar medium and incubated at 37°C for 24 hours. The MIC was taken to be the lowest concentration inhibiting the growth of the organisms.

## Results

Medicinal plants were screened *in vitro* for vibriocidal activity against 12 isolates of *Vibrio cholerae* non-O1, and one standard strain each of *Vibrio cholerae* and *Vibrio parahaemolyticus*. The vibriocidal activity of 48 extracts (aqueous, acetone, and ethanol) belonging to 16 plant species were investigated [Table 2]. The MIC values of active extracts containing the potential bioactive compound were also determined [Table 3]. Among the plants tested *Syzygium cumini, Saraca indica, Terminalia belerica, Datura stramonium, Lawsonia inermis,* and *Allium sativum* showed high vibriocidal activity. The range of MIC values in the ethanolic extracts of *Syzygium cumini, Lawsonia inermis, Terminalia belerica,* and *Magnifera indica* were found to be 2.5–20 mg/ml, 2.5–10 mg/ml, 2.5–20 mg/ml, and 15–20 mg/ml, respectively, against test bacteria. The range of MIC values in the aqueous extracts of *Allium sativum, Azadirachta indica, Tamarindus indica, Punica granatum, Eugenia caryophyllus, Mimusops elengi,* and *Piper nigrum* were found to be 5–15 mg/ml, 5–20 mg/ml, 10–15 mg/ml, 5–20 mg/ml, 15–20 mg/ml, 15–20 mg/ml, and 20 mg/ml, respectively, against test bacteria. The range of MIC values in the acetone extracts of *Saraca indica, Datura stramonium, Madhuca latifolia, Acacia catechu,* and *Acacia arabica* were observed as 2.5–10 mg/ml, 2.5–15 mg/ml, 10–20 mg/ml, 10–20 mg/ml, and 10–20 mg/ml, respectively, against test bacteria. The MIC values in ethanolic extracts were a minimum, especially against *Vibrio cholerae, Vibrio cholerae* non-O1, and *Vibrio parahaemolyticus* (2.5 mg/ml). The MIC values in the aqueous extracts of *Allium sativum* were a minimum for both *Vibrio cholerae* and *Vibrio parahaemolyticus* (2.5 mg/ml) and eight isolates of *Vibrio cholerae* non-O1 (5 mg/ml). The MIC values in the acetone extracts of *Saraca indica* and *Datura stramonium* were low for *Vibrio cholerae* (2.5–5 mg/ml), *Vibrio parahaemolyticus* (2.5 mg/ml), and four isolates of *Vibrio cholerae* non-O1 (2.5–5 mg/ml). Extracts of *Eugenia caryophyllus, Mimusops elengi, Piper nigrum, Punica granatum,* and *Tamarindus indica* showed poor vibriocidal activity.

**Table 2:** Vibriocidal activity (inhibition zone in mm) of plant extracts against test bacteria

*Plants*	*Solvent*	*BGCC No.*													
		
		*V. ch.*	*V. para.*	*59*	*60*	*61*	*62*	*63*	*64*	*65*	*66*	*67*	*68*	*69*	*70*
*Syzygium cumini*	Water	10	10	8	7	11	11	9	13	-	9	12	11	11	9
	Acetone	10	11	13	11	11	9	9	10	8	-	-	9	8	12
	Ethanol	11	12	18	22	15	12	18	21	19	13	16	9	12	11
*Lawsonia inermis*	Water	11	10	11	15	9	11	8	12	-	10	9	-	-	9
	Acetone	15	12	20	19	14	19	20	28	9	16	13	12	11	12
	Ethanol	22	17	17	20	15	20	21	17	12	21	15	20	17	19
*Terminalia bellerica*	Water	12	10	10	15	12	14	12	16	12	11	15	15	11	11
	Acetone	14	14	10	15	15	13	17	17	14	14	13	14	15	13
	Ethanol	24	21	3	15	15	-	17	16	15	15	21	20	-	-
*Saraca indica*	Water	13	11	15	17	19	14	12	12	12	10	13	14	12	11
	Acetone	20	14	15	16	17	20	13	16	14	19	24	19	16	23
	Ethanol	13	11	12	14	13	11	9	11	13	10	14	9	11	11
*Datura stramonium*	Water	12	11	13	22	15	22	18	15	12	12	12	14	14	-
	Acetone	16	14	14	15	17	15	17	19	15	13	20	19	14	9
	Ethanol	12	10	11	12	13	14	13	14	9	10	14	13	12	7
*Allium sativum*	Water	19	16	22	14	23	21	13	-	-	13	17	15	22	17
	Acetone	18	15	21	17	20	18	24	-	-	-	22	27	12	16
	Ethanol	16	11	15	12	16	15	10	9	12	-	16	9	15	14
*Madhuca latifolia*	Water	9	10	6	8	9	9	8	-	12	12	9	11	10	10
	Acetone	11	13	7	9	11	11	9	-	10	11	10	11	11	10
	Ethanol	11	10	-	-	9	10	8	7	9	10	11	10	9	9
*Acacia catechu*	Water	9	7	6	6	9	8	8	-	-	-	8	8	8	9
	Acetone	11	9	7	7	10	10	9	-	9	8	10	11	10	10
	Ethanol	10	8	-	-	9	10	7	8	9	8	9	10	9	10
*Acacia arabica*	Water	9	10	7	10	10	9	8	-	11	11	8	13	10	10
	Acetone	11	10	7	11	11	14	10	8	10	9	11	14	13	11
	Ethanol	10	9	-	9	10	12	9	-	10	7	10	9	10	9
*Azadirachta indica*	Water	-	-	13	-	15	12	10	14	-	-	11	9	12	9
	Acetone	12	10	-	11	9	11	8	9	7	-	-	7	7	-
	Ethanol	10	9	10	8	9	-	9	-	-	-	9	-	8	-
*Tamarindus indica*	Water	12	10	14	-	-	12	-	-	-	10	-	13	11	-
	Acetone	-	-	-	-	-	-	-	-	-	-	-	-	-	-
	Ethanol	-	-	-	-	-	-	-	-	-	-	-	-	-	-
*Punica granatum*	Water	13	11	12	17	-	7	-	7	19	12	-	10	-	-
	Acetone	11	9	12	-	-	-	-	7	-	10	-	-	-	-
	Ethanol	9	10	10	9	-	-	-	-	11	9	-	9	-	-
*Eugenia caryophyllus*	Water	10	-	-	-	-	-	-	10	-	-	-	-	-	-
	Acetone	12	10	10	12	16	-	15	13	9	-	11	-	7	-
	Ethanol	-	-	8	-	11	-	-	10	-	-	9	-	-	-
*Mimusops elengi*	Water	10	7	10	7	13	9	9	8	8	9	8	-	10	7
	Acetone	-	-	-	-	-	-	-	-	-	-	-	-	-	-
	Ethanol	-	-	-	-	-	-	-	-	-	-	-	-	-	-
*Magnifera indica*	Water	-	-	-	-	-	-	-	-	-	-	-	-	-	-
	Acetone	-	-	-	-	-	-	-	-	-	-	-	-	-	-
	Ethanol	11	9	10	8	10	14	14	9	10	10	9	-	-	10
*Piper nigrum*	Water	9	-	-	10	11	7	-	-	-	8	7	11	-	10
	Acetone	-	-	-	-	-	-	-	-	-	-	-	-	-
	Ethanol	-	-	-	-	-	-	-	-	-	-	-	-	-	-

V. ch. = standard strain of *Vibrio cholerae* (H17004), V. para = standard strain of *Vibrio parahaemolyticus* (KX-V138), 59 – 70 = isolates of *V. cholerae* non-O1, (-) = no activity, BGCC = Bacterial Germplasm Culture Collection.

**Table 3:** Minimum inhibitory concentration (mg/ml) of plant extracts against the test bacteria

Plant Name	BGCC No.													
	
	*V. ch.*	*V. para*	*59*	*60*	*61*	*62*	*63*	*64*	*65*	*66*	*67*	*68*	*69*	*70*
*Syzygium cumini*	10	10	5	5	10	10	5	10	2.5	10	5	20	10	15
*Lawsonia inermis*	2.5	2.5	2.5	5	2.5	5	5	10	5	2.5	5	2.5	2,5	2.5
*Terminalia bellerica*	2.5	2.5	15	5	2.5	15	10	2.5	2.5	10	10	2.5	15	20
*Saraca indica*	2.5	5	2.5	2.5	2.5	2.5	10	2.5	10	5	2.5	10	5	2.5
*Datura stramonium*	2.5	2.5	15	15	5	2.5	5	5	5	5	2.5	5	10	15
*Allium sativum*	10	10	5	5	5	10	10	5	10	15	5	5	5	5
*Madhuca latifolia*	15	15	20	20	15	15	15	15	10	10	15	15	15	10
*Acacia catechu*	15	15	20	20	10	15	15	20	20	20	15	10	15	15
*Acacia arabica*	15	15	20	15	15	10	15	20	15	20	15	10	10	15
*Azadirachta indica*	15	10	15	-	5	15	15	10	-	-	15	20	10	20
*Tamarindus indica*	15	15	10	-	-	10	-	-	-	15	-	10	10	-
*Punica granatum*	10	10	10	10	-	20	-	20	5	10	-	15	-	-
*Eugenia caryophyllus*	15	20	15	15	15	-	15	20	20	-	15	-	20	-
*Mimusops elengi*	15	20	15	20	15	20	20	20	20	20	20	-	15	20
*Magnifera indica*	15	20	15	20	15	15	15	20	15	15	20	-	-	15
*Piper nigrum*	20	20	-	20	20	20	-	-	-	20	-	20	20	-

V. ch. = standard strain of *Vibrio cholerae* (H17004), V. para = standard strain of *Vibrio parahaemolyticus* (KX-V138), 59 – 70 = isolates of *V. cholerae* non-O1, (-) = no activity, BGCC = Bacterial Germplasm Culture Collection.

## Discussion

The aqueous, ethanolic, and acetone extracts exhibited potent vibriocidal activity against the bacterial strains involved in the study. The present study reports the vibriocidal activity of plant extracts used by tribals in Indian folklore medicine against cholera and gastrointestinal infections. The antibacterial activity of *Lawsonia inermis* has been reported against *Bacillus cereus, Bacillus anthracis, Eschirichia coli,* and *Staphylococcus aureus*.[14] The methanolic extract of *Tamarindus indica* has been reported to inhibit the growth of *Burkholderia pseudomallei,* having a clinical origin. The MIC value of the extracts of *Tamarindus indica* leaves against *Burkholderia pseudomallei* was reported as 125 μg/ml.[15] Similar results have been obtained in the present study. The vibriocidal action of the different extracts from the leaves of the shrub of *Punica granatum* against various strains of *Vibrio cholerae* has been reported.[6] The presence of antibacterial activity in the leaf extract of *Mimusops elengi* against *V. cholerae* non O1 has been determined in this study. Similar results were reported by Satyanarayana *et al.*[7] Mathabe *et al.*,[16] studied the methanolic, ethanolic, acetone, and aqueous extracts from different extracts of *Indigofera daleoides, Punica granatum, Syzygium cordatum, Gymnosporia senegalensis, Ozoroa insignis, Elephantorrhiza elephantine, Elephantorrhiza burkei, Ximenia caffra, Schotia brachypetala,* and *Spirostachys Africana,* to determine the presence of antibacterial activity against *Vibrio cholerae, Escherichia coli, Staphylococcus aureus, Shigella* spp., and *Salmonella typhi.* The extracts from the different plants showed relatively high antibacterial activity against most of the tested microorganisms with the diameter of inhibition zones ranging between 10 to 31 mm.

Modern and traditional healthcare often exist side by side, but seldom cooperate. The reasons are, lack of standardization in traditional medicine with respect to raw materials, method of production, and quality control of the finished product.[17] The use of traditional medicine often relies on mysticism and intangible forces such as witchcraft, with some aspect based on spiritual and moral principles. While these may be valid psychologically, they cannot be rationalized scientifically.[18] From the results it can be concluded that plant extracts have a great potential as antimicrobial compounds against microorganisms, and therefore, can be used in the treatment of infectious diseases caused by resistant microorganisms. Since the aqueous extract of *A. sativum* and the acetone extracts of *S. indica* and *D. stramonium* showed MIC of 2.5–5 mg/ml, these plants can be used to identify bioactive natural products, which may serve as leads for the development of new pharmaceuticals that can address the therapeutic need.

## References

[1] World Health Organization

[2] Albert MJ, Siddique AK, Islam MS, Faruque AS, Ansaruzzaman M, Faruque SM (1993). Large outbreak of clinical cholera due to *V. cholerae* non-O in Bangladesh. Lancet.

[3] Monroe S, Polk R (2000). Antimicrobial use and bacterial resistance. Curr Opin Microbiol.

[4] Diallo D, Hveem B, Mahmoud MA, Betge G, Paulsen BS, Maiga A (1999). An ethanobotanical survey of herbal drugs of Gourma district, Mali. Pharma Biol.

[5] Edeoga HO, Okwo DE, Mbaebie BO (2005). Phytochemical constituents of some Nigerian medicinal plants. Afr J Biotech.

[6] Das A, Mohapatra SB, Mishra RK, Pal BB (2001). *In vitro* vibriocidal activity of *Punica granatum*. Phytomed.

[7] Satyanarayana T, Rao DPC, Singh BS (1977). Antibacterial activity of six medicinal plant extracts. Indian Drugs.

[8] Muruganandan S, Srinivasan K, Chandra S, Tandan SK, Lal J, Raviprakash V (2001). Anti-inflammatory activity of *Syzygium cumini* bark. Fitoterapia.

[9] Reddy SV, Pullela VS, Praveen B, Hara Kishore K, Suryanarayana Murthy U, Rao MJ (2004). Antibacterial constituents from the berries of *Piper nigrum*. Phytomed.

[10] Rojas JJ, Ochoa VJ, Ocampo SA, Muñoz JF (2006). Screening for antimicrobial activity of ten medicinal plants used in Colombian folkloric medicine: A possible alternative in the treatment of non-nosocomial infections. BMC Compl and Alterna Med.

[11] Collins CH, Lynes PM, Grange JM (1995). Microbiological methods.

[12] Nair R, Kalariya T, Chanda S (2005). Antibacterial activity of some selected Indian medicinal flora. Turk J Biol.

[13] Rios JL, Recio MC, Villar A (1988). Screening methods for natural products with antimicrobial activity: A review of the literature. J Ethnopharmacol.

[14] Malekzadeh F (1968). Antimicrobial Activity of *Lawsonia inermis* L. Appl Microbiol.

[15] Muthu SE, Nandakumar S, Rao UA (2005). The effect of methanolic extract of *Tamarindus indica* L. on the growth of clinical isolates of *Burkholderia pseudomallei*. Indian J Med Res.

[16] Mathabe MC, Nikolova RV, Lall N, Nyazema NZ (2006). Antibacterial activities of medicinal plants used for the treatment of diarrhoea in Limpopo Province, South Africa. J Ethnopharmacol.

[17] Anand N (1984). Integrated approach to development of new drug from plants and indigenous remedies. In Natural products and drug development.

[18] Iwu MM (1994). African medicinal plant in the search for the new drugs based on ethanobotanical leads.

